# Examining the endpoint impacts, challenges, and opportunities of fly ash utilization for sustainable concrete construction

**DOI:** 10.1038/s41598-023-45632-z

**Published:** 2023-10-25

**Authors:** Christian Orozco, Somnuk Tangtermsirikul, Takafumi Sugiyama, Sandhya Babel

**Affiliations:** 1 Sirindhorn International Institute of Technology, Thammasat University, Pathum Thani, 12120 Thailand; 2 Graduate School of Engineering, Hokkaido University, Sapporo, Hokkaido 060-8628 Japan; 3 Faculty of Engineering, Hokkaido University, Sapporo, Hokkaido 060-8628 Japan

**Keywords:** Ecology, Environmental sciences, Environmental social sciences

## Abstract

Fly ash has been widely used as a cement substitute to improve the sustainability of concrete. Although the advantages of fly ash have been extensively documented, there is a gap in understanding why its use in mass concrete applications remains low in some countries, such as the Philippines. Thus, this work aims to understand the issues that impede waste utilization, particularly fly ash in the concrete construction industry, quantify the impact of the current practice, and identify opportunities for sustainable fly ash utilization. Endpoint impact analysis was conducted through the life cycle using SimaPro 9.3 to quantify the impacts on human health, ecosystem, and resources of 31 concrete mixtures of low, normal, and high strength design with 0 to 20% fly ash as cement replacement. In-depth, semi-structured interviews with key stakeholders were undertaken to determine the institutional, economic, social, and technological challenges related to the utilization of waste materials in large-scale concrete construction. More than 90% of the total impact of concrete contributes to damage to human health, primarily caused by global warming and fine particulate matter. The use of fly ash at 20% replacement by weight of cement benefits resources more significantly than human health and the ecosystem. The use of chemical admixture to improve strength has a significant impact on resources. High fly ash replacement for normal and high-strength concrete has a greater reduction in all endpoint categories than for low-strength design. Recommendations are proposed to maximize the beneficial impact of using fly ash in the concrete industry.

## Introduction

Concrete is the world’s most consumed manufactured construction material^1,2^. As a popular construction material, the concrete industry will continue to play a significant role in global construction for an extended period^3^. Concrete’s vast production and consumption cycles have a severe environmental impact^4^, rendering the present industry unsustainable. It is critical for the concrete industry to develop strategies for the mitigation of environmental and societal impacts. One approach to achieving tangible sustainability is using waste materials as a substitute for the conventional components of concrete^5,6^. Various cement replacements are sourced from industrial by-products and agricultural and municipal solid wastes. The industrial by-products that have been utilized in place of using cement in concrete are silica fume^7^, coal fly ash^8^, bottom ash^9^, granulated blast-furnace slag^10^, and limestone powder^11^. Agricultural ashes from rice husks and palms are also used^12,13^. The most commonly utilized by-product as cement replacement is fly ash due to its magnitude, compatibility with cement, and relatively low cost^14^. It is a coal combustion by-product, accounting for a least 85% of the ash generated in coal-fired power plants^15^. However, the global average usage of fly ash is just 53.5% of the total output^16^. In 2008, it was estimated that the world produced 777 million tons of fly ash^12^. Fly ash can potentially improve the compressive strength and segregation of concrete^17^ as well as its many durability properties^8,18^. The use of fly ash as a cement replacement will minimize landfill disposal.

Several studies have utilized the life cycle analysis (LCA) approach to estimate the benefits of employing fly ash as a building material^19,20^. Since 1990, LCA has been used in the construction sector as a critical technique for assessing the environmental effect of materials and the environmental performance of buildings^21,22^, as well as for evaluating various construction materials^23,24^. Vieira et al.^25^ performed an environmental assessment of fly ash and slag utilization in self-compacting concrete (SCC), considering strength and service life, and concluded that using slag is preferable. In addition, Celik et al.^26^ showed that 55% fly ash replacement by weight of cement creates highly workable SCC mixtures with low global warming potential for concrete manufacture. In comparison with concrete made with pure OPC, Chen et al.^27^ discovered that applying fly ash on pervious concrete displayed the highest overall performance in balancing several criteria, including cost, material properties, greenhouse house gas reduction, and energy. Using LCA, Hafez et al.^28^ concluded that transporting fly ash from China to the United Kingdom negates the environmental advantages of its replacement of cement. The study of Radwan et al.^20^ demonstrates that 50% and 70% OPC substitution will lower the impact by an average of 44% and 61%, regardless of the blending scheme of fly ash and/or slag.

Life cycle impact assessments are typically conducted at the midpoint and endpoint levels. Notably, previous research in the field of impact assessment of concrete has predominantly focused on midpoint impacts^20,26,29^. The selection between midpoint and endpoint methods for impact assessment depends on considerations of relevance and reliability^30^. In this study, preference is given to the endpoint method due to its greater relevance, as it allows to compare the different options investigated. Endpoint impact assessment has been used in the assessment of concrete gravity dams^31^ and concrete with recycled aggregates^32^. The endpoint method facilitates the generation of comparable single overall environmental score and damage-focused indicators, which can easily be understood by decision-makers^21^. Endpoints are often components that the public believes should be protected^33^.

Although several literatures have documented the advantages of fly ash^8,34,35^, there exists a gap in understanding why the utilization of fly ash in mass concrete applications remains low in some countries. According to the Philippine Department of Energy, the five biggest coal-fired power plants in the Philippines generate around 1.4 million tons of ash annually, with relatively poor utilization^36^. This is anticipated to increase as additional coal-fired power plant facilities are constructed nationwide. Fly ash that is not used is disposed of in ash ponds and landfills^37^. However, due to the metal and mineral composition of coal ash, its disposal poses a risk of surface and groundwater contamination^38^. The construction sector can use this massive waste resource^39^. It is important to understand the barriers that restrict the widespread adoption of these materials as a cement replacement in the concrete construction industry to maximize the use of this waste resource.

The aim of this work is to (1) quantify the impacts/benefits of fly ash utilization on human health, ecosystem, and resources, (2) explore the challenges that impede waste utilization in the concrete construction sector, particularly fly ash, and (3) identify opportunities for sustainable utilization of fly ash in mass concrete production. In this study, we employed both quantitative and qualitative methods. Quantitatively, we assessed the sustainability impact of current concrete production through a life cycle endpoint analysis. Qualitatively, we conducted key stakeholder interviews to identify barriers and motivations, aiming to develop a comprehensive roadmap for the future of sustainable concrete consumption. This study will contribute to existing fly ash concrete literature by unraveling the impacts of fly ash utilization on reducing the impact of mass concrete associated with human health, ecosystem, and resources. The study result will benefit countries with similar conditions and concrete industries with similar practices.

## Methodology

**Figure 1:**
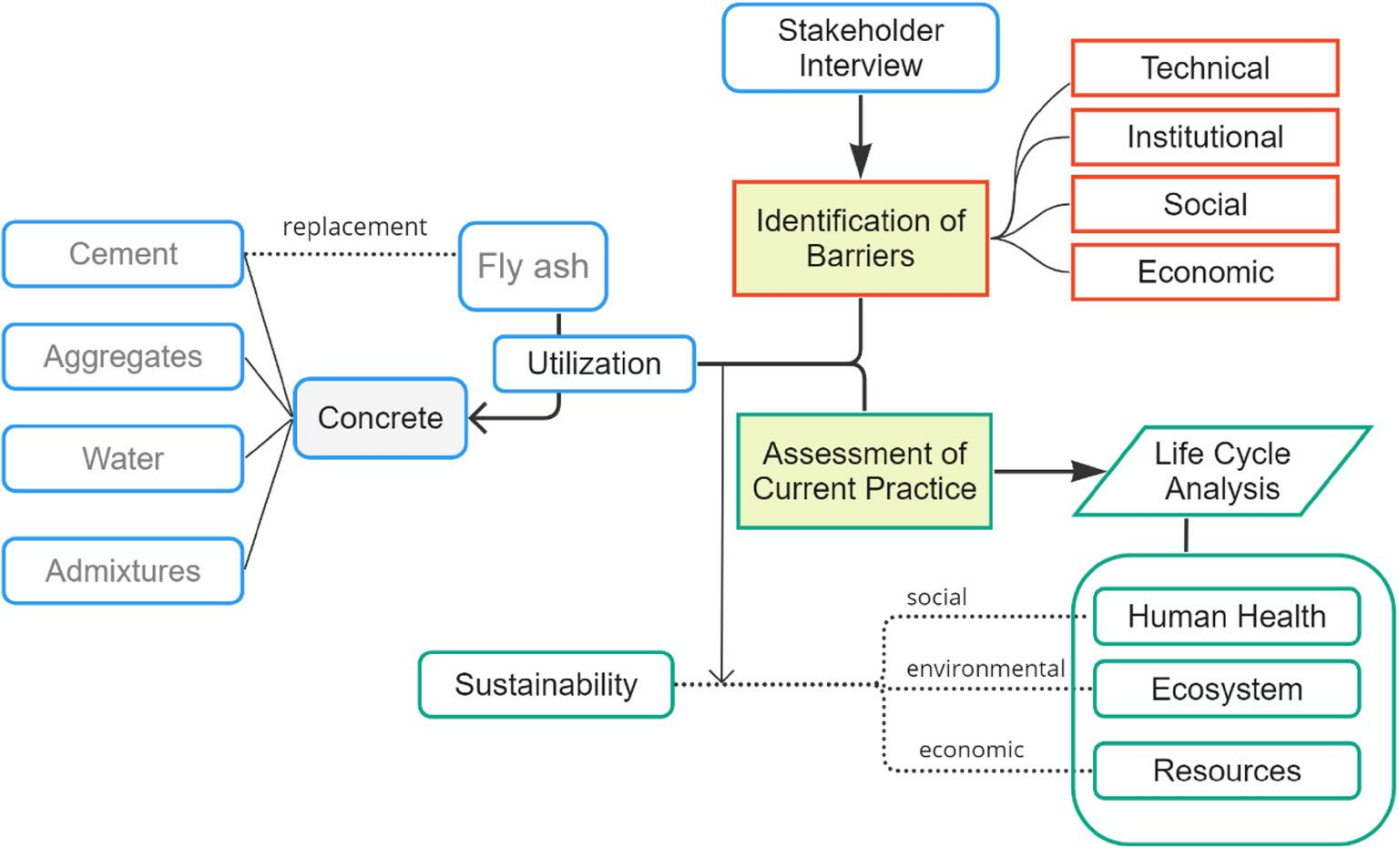
The methodological framework of the study.

Two major components are included in the general framework (Fig. 1) used in this study: (1) a stakeholder interview to understand the motivation/barrier for using waste material in the concrete construction industry, and (2) an impact assessment of the current use of waste materials, specifically fly ash, in mass concrete applications using life cycle endpoint analysis. By understanding the barriers and quantitatively assessing the sustainability dimension of the current practice, a comprehensive formulation of a framework for more sustainable utilization of fly ash is developed. In addition, an analysis of the institutional, economic, social, and technological barriers related to the utilization of waste materials in actual practice is carefully documented and analyzed.

### Stakeholder interview

Interviews with key stakeholders were conducted to understand the motivation and barriers to using cement replacing material in the Philippine concrete construction industry, specifically, but not limited to, fly ash. The motivations and barriers to waste utilization in concrete construction were qualitatively identified using the interview structure developed by Henry and Kato^40^ with minor adjustments. In-depth, semi-structured interviews were conducted with a diverse group of key stakeholders with decades of expertise in the concrete building industry. The semi-structured interview adhered to a general structure but allowed for an in-depth discussion of individual issues. The interview covers a wide range of issues, including specific industry conditions in the Philippines, current waste material use, and the possibilities and constraints of large-scale waste material use (institutional, economic, social, and technological). The specific questions posed to the stakeholders are listed in Supplementary Table S1. A combination of individual online and offline interviews and focus group discussions were conducted. Given the nature of our research objectives and the flexibility provided by semi-structured interviews, conducting these interviews both online and offline would yield similar results. Ten interviews were conducted in all. The interviewees have a minimum of 10 years of experience coming from various industries within the concrete construction industry. The interviews were conducted following relevant ethical guidelines and regulations in the Philippines, and all subjects gave their informed consent. Table 1 highlights the stakeholders that were interviewed with their backgrounds. The respondents were chosen because they had specific experience in concrete development, use, and management, which allowed them to identify the issues in the national context. The analysis of the interview data was conducted using the SWOT (Strengths, Weaknesses, Opportunities, and Threats) and a modified PEST (Political, Economic, Social, and Technological) technique, which enabled us to organize the findings into structured points (social and institutional, economic, technical).

**Table 1:** Background of expert stakeholders interviewed.

Stakeholders in the Philippines	Number	Years of experience
Concrete consultant	2	40+
Contractor	3	20–30
Concrete testing manager	1	30+
Academe	2	10+
Batching plant owner	2	10+

### Life cycle assessment

This study employs the International Organization for Standardization (ISO) 14040 standard for LCA, which has been used in earlier investigations^41,42^. It is a well-established tool for the environmental assessment of different concrete mixtures^43^. The SimaPro 9.3 LCA program and the EcoInvent 3.8 database were used to evaluate the concrete mixtures. SimaPro has been extensively utilized in the LCA of concrete and cement^44,45^. On the other hand, EcoInvent was recently regarded as one of the finest construction materials databases^46^. The life cycle study was conducted with local conditions in the Philippines. To assess the influence of fly ash on various concrete mix designs, a cradle-to-gate life cycle analysis of various concrete mixtures was made, similar to many previous studies^42,47^.

#### Concrete mix designs

This research examines the impact of fly ash utilization on a wide range of concrete mixtures. The data were obtained from two batching plants located in the capital region of the Philippines. This includes 31 concrete mix designs covering low, normal, and high-strength concrete mixtures. The mixtures obtained from concrete batching plants used for mass concrete applications have a range of 2000 psi (13.8 MPa) to 10,000 psi (69.0 MPa). Concrete can be categorized into the following groups based on its 28-day compressive strength: low strength for concrete with a compressive strength below 20 MPa, normal strength for concrete with a compressive strength between 20 and 40 MPa, and high strength for concrete with a compressive strength above 40 MPa. In the Philippines, 3000 psi (20.7 MPa) or less is considered low strength, while 6000 psi (41.4 MPa) or higher is considered high strength for mass concrete applications. Table 2 indicates the number of concrete mixtures acquired per class of concrete, including their typical applications. The mix proportions, which include the amount of cement, sand, gravel, water, admixture, and fly ash, can be found in Supplementary Table S2.

**Table 2:** Number of concrete mixtures analyzed and their applications.

Fly ash replacement	0%	10%	15%	20%	Typical applications
Low strength (13.8–20.7 MPa)	2	2	1	2	Elements of a typical residential structure, low-traffic pavement
Normal strength (24.3–34.5 MPa)	3	3	3	3	House slabs (ground slabs), columns and beams for residential applications, rigid pavements, bridge decks, suspended slabs, walls (including shear walls), and most mid-rise structures (up to 15 stories)
High Strength (41.4–69.0 MPa)	4	4	3	1	Most high-rise structures (up to 20 stories), elevated transport infrastructures, special infrastructures with high load

The concrete mixtures investigated in this study incorporate varying percentages of fly ash as partial replacements for cement, specifically 0%, 10%, 15%, and 20%. These replacement levels align with the typical practices observed in the Philippine concrete construction industry. The fly ash utilized is sourced from coal-fired power plants in the Philippines and is mixed into the concrete as a mineral admixture at the concrete production plants. It is important to note that the fly ash is obtained from third-party suppliers. The specific type of fly ash employed in the batching plants is classified as Class F fly ash according to ASTM C618. The chemical composition of this fly ash includes a CaO content of 10.6%, SiO_2_ content of 53.1%, Al_2_O_3_ content of 17.5%, and Fe_2_O_3_ content of 5.48%. The loss on ignition (LOI), as determined through testing conducted in accordance with ASTM C311, is recorded as 2.7. The provided chemical composition details were obtained directly from the fly ash supplier.

#### Scope, functional unit, and life cycle inventory

**Figure 2:**
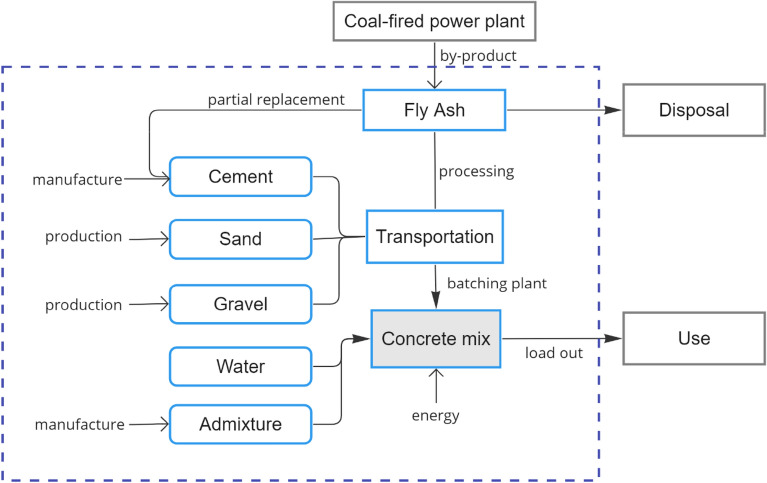
System boundary used in life cycle assessment of mass concrete production.

The functional unit selected for this study is one cubic meter of concrete, a widely adopted measure in numerous Life Cycle Assessment (LCA) investigations concerning concrete^42,48^. The system boundary, as depicted in Fig. 2, encompasses all the processing inputs necessary for the production of concrete. This study focuses on the principal manufacturing processes involved in concrete constituents, considering the complete life-cycle stages encompassing raw material extraction, transportation, and mixing operations conducted at a ready-mix plant.

The system boundary adopted in this study encompasses the complete life cycle of cement and aggregates, including their extraction, processing, and transportation to batching plants. It should be noted that fly ash is a by-product of industrial activities, and thus, this study does not specifically evaluate the consequences associated with fly ash production. However, certain aspects related to fly ash preparation, such as minimal drying processes, were taken into consideration. Particular attention was given to transportation activities, as they are recognized as significant contributors to emissions associated with fly ash. The end-of-life strategy for concrete is beyond the scope of this investigation. Given that the concrete mixtures under examination were designed to meet similar strength requirements and functional specifications, factors related to the construction, service, and demolition stages were deemed to have no direct influence on the findings of this study.

Cement is the primary ingredient of concrete. Local data for cement production were obtained from comprehensive Environmental Impact Assessment Reports of two major cement factories in the Philippines. The grinding process consists of 90% clinker, 5% gypsum, and 5% mineral filler. The information used to describe Portland cement production is based on the American Society for Testing and Materials’ technical standard (ASTM C150) for Portland cement. The Philippines’ local cement standard adopts the ASTM Standard. The dataset utilized for sand transformation describes a blend of riverbed and quarry sand. According to interviews with operators of ready-mix batching plants, roughly 80% of the aggregates come from riverbeds, and 20% are from quarries. For gravel, the dataset represents the operation of open-pit mechanized mining. Drilling, blasting, separation, and collecting are carried out on the mining site. The utilized chemical additives are superplasticizers. For admixture, worldwide data was utilized because most Philippine admixtures are imported from overseas. Type G polycarboxylate admixtures conforming to ASTM C494 are the most frequently used admixtures in the country. Global average data or GLO were used in Ecoinvent for the emission caused by this plasticizer. The electricity utilized is medium voltage Philippine electrical market. At the batching plant, 76–78 kWh/m^3^ of energy is required for concrete mixing^49^.

The transportation of cement-replacement materials in the context of life cycle analysis is a critical component of this study. In this investigation, all the examined components are transported via truck, with weights ranging from 16 to 32 tons. To estimate the transportation data, the latitude of the concrete batching plant’s suppliers (i.e., material sources) is utilized, and the land route is calculated using a geographical information system. The data obtained represents the average distances between the sources and the batching plants situated within the metropolitan area. The distances considered for the various materials are as follows: 150 km for cement, 121 km for fly ash, 36 km for gravel, and 100 km for sand. The presence of on-site water sources eliminates the necessity for water transportation. The mode of transport used in the EcoInvent database is specified as “transport freight lorry 16–32 metric tons euro 3,” and the geographical data for the rest of the world (RoW) is employed.

#### Endpoint analysis

The impact analysis was conducted using the latest, state-of-the-art 2016 version of ReCiPe. Due to its broad application in numerous scientific models, the consensus model, also known as the Hierarchist model of ReCiPe, is utilized in this study. This approach is based on widely recognized timeframe and other factors^48^. The midpoint and endpoint impact analyses are the two most common assessment techniques used to determine the influence of concrete mixtures. The endpoint technique has advantages since it produces simple outcomes by evaluating damages to human health, the ecosystem, and resources (see Supplementary Fig. S1). For the human health category, ReCiPe employs disability-adjusted life years (DALY), which represent a life lost in years or damaged due to environmental impacts. The ecosystem damage category is assessed by species/year; this represents species lost in a year because of emissions to the environment, water body, etc. The resource damage category is based on economic loss due to a marginal rise in costs because of resource scarcity. The cost associated with this resource damages is expressed in equivalent 2013 U.S. Dollars^50^. Midpoint and endpoint approaches are complementary. However, the endpoint characterization provides better information on the environmental relevance of the environmental flows. Moreover, the contribution of concrete is greater at the endpoint level than at the midpoint^51^.

#### Statistical analyses

The paired t test was employed to assess the significance of differences in the impacts (damages) among concrete mixtures. This statistical analysis method allows for the determination of whether the mean difference between two sets of observations is statistically significant. In the context of this study, the t test is employed to examine whether the substitution of cement with fly ash has a substantial impact on reducing the associated endpoint impact across concrete mixtures. Endpoint impacts of concrete mixtures with similar compressive strength design, but different fly ash content is considered data pairs. The interpretation of the test results is based on a 95% level of confidence or a 5% level of significance. Under this threshold, a difference between the impacts is deemed significant if the *p* value is less than 0.05.

In addition, correlation analysis is conducted to discern the influence of specific concrete components on the endpoint damage indicators related to human health, ecosystem, and resources. Correlation analysis is a bivariate examination that evaluates the strength and direction of the association between two variables. Spearman’s correlation coefficient (*ρ*) is utilized in this study to explore the monotonic relationship between continuous or ordinal variables. The correlation coefficient can range from + 1 to −1, with its value indicating the strength of the association. A coefficient closer to + 1 indicates a stronger positive correlation between the variables. Spearman correlation provides valuable insights into the impact of concrete components on endpoint indicators, especially when prior knowledge of the relationship is limited. This method of analysis has also been used in a previous study on life cycle analysis of concrete^52^. The statistical software Minitab 21 was used to perform all statistical analyses.

### Ethics and inclusion statement

All methods were carried out in accordance with relevant guidelines and regulations. The data gathering with stakeholders was conducted in accordance with the Data Privacy Act of the Philippines (Republic Act No. 10173). All subjects gave their informed consent for inclusion.

## Results and discussion

The following sections discuss the results of endpoint impacts and key stakeholder interviews.

### Endpoint analysis: impacts on human health, ecosystem, and resources

**Figure 3:**
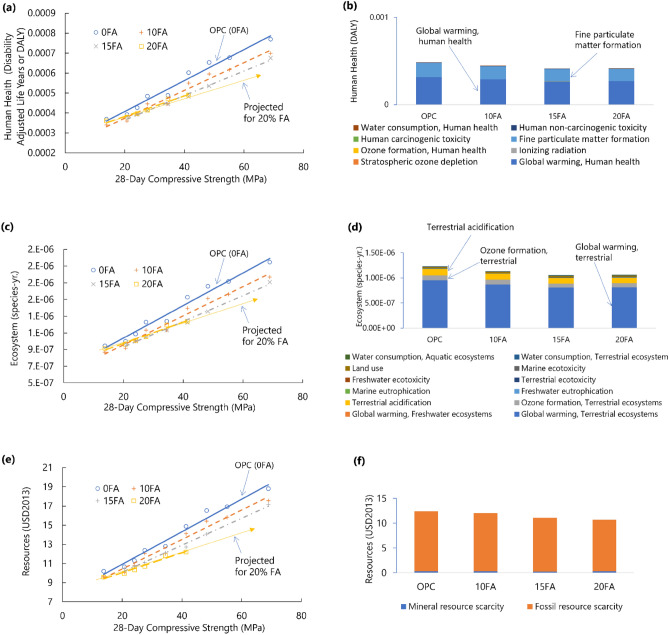
Endpoint impacts on (**a**) human health, (**c**) ecosystem, and (**e**) resources of 31 concrete mixtures with 0%, 10%, 15%, and 20% fly ash as cement replacement. Significant midpoint impact indicators contributing to endpoint impacts of (**b**) human health, (**d**) ecosystem, and (**f**) resource damage for 27.6 MPa concrete.

The effects of using different fly ash replacements on human health for different concrete mix designs are illustrated in Fig. 3a and Supplementary Table S3. When utilizing 10%, 15%, and 20% fly ash, the reduction is 7.9–8.7%, 2.6–19.9%, and 2.6–18.7%, respectively, when compared to pure-cement concrete. The paired *t* test comparison (Supplementary Tables S6 and S9) with pure OPC concrete indicates a significant difference (reduction in human health damage) at 95% confidence for 10% and 15% (*p* < 0.05) fly ash replacement. By substituting 20% fly ash for cement, the impact on human health falls dramatically at a 10% level of significance. This indicates that the use of fly ash will significantly lessen the negative effects of concrete production on human health. The extent of human health damage caused by global warming (65.12%) and fine particulate matter generation (33.44%) for a 27.6 MPa concrete, one of the most widely used concrete mix in the Philippines, is shown in Fig. 3b. Overall, these two categories account for more than 98% of all human health damage. Global warming is a major environmental concern, and the role of concrete in CO_2_ production has been widely discussed in scientific literature^53^. However, the influence of fine particulate matter should be examined as well. This is because studies have shown that tiny particles cause more life years to be lost. According to the findings of this investigation, fine particulate matter accounts for 33.44% of the total contribution by a 27.6 MPa concrete. Dust contributes considerably to PM_10_ and PM_2.5_, particularly as a result of cement manufacture, ready-mix facilities, and limestone mining^54^. Fine particulate matter is a critical health issue that needs to be addressed because it is a major source of the country’s air pollution levels. Current concentrations of PM_2.5_ in urban regions of the Philippines exceed the guidelines of the World Health Organization (WHO), according to published studies^55,56^. Air pollution continues to have a devastating impact on the health of Filipinos. Traffic enforcers in Metro Manila, for instance, are 124 times more likely to develop chronic obstructive pulmonary disease if continuously exposed to high PM_2.5_ concentrations^56^. Reduced cement use by replacing fly ash in manufacturing concrete batching plants will significantly minimize the generation of fine particulate matter by reducing the fly ash disposed of in landfills, as well as the emissions generated during cement production.

The influence of utilizing fly ash as a replacement for cement on ecosystem damage (Fig. 3c) follows a similar pattern (Supplementary Tables S4 and S7) to the results obtained from human health (Fig. 3a). Considering a 27.6 MPa concrete, global warming is the primary cause of environmental damage (77%) and consequently to human health due to climate change (Fig. 3d). Terrestrial acidification (10.28%), ozone formation (7.97%), and land use (2.29%) are the other major drivers of ecological destruction, as shown in Fig. 3d. Other factors, such as eutrophication, ecotoxicity, and water use, have little impact on ecosystem damage. Terrestrial acidification is a global hazard to plant diversity, mainly produced by NO_x_, NH_3_, and SO_2_, which are anthropogenic atmospheric pollutants^57^. This causes acidification of rivers/streams and soil. All these harmful gases are released during the production of cement. In the Philippines, sustainability initiatives are yet to be incorporated into cement production. Acidification causes the mobilization of heavy metals and soil leaching, which has an adverse effect on terrestrial and aquatic animals^58^, as well as plants, by disturbing the food chain. The main factors influencing acidification are NO_x_ and SO_2_ in air and H_2_S and H_2_SO_4_ in water systems^59^. The combustion of diesel fuels mainly generates ozone during the different stages of concrete’s life cycle. Other midpoint impacts account for less than 3% of total ecological damage. The impact of using fly ash at various 28-day compressive strengths on the ecosystem is similar to that of the impact on human health.

The environmental advantage of employing fly ash is particularly obvious in the domain of resource damage reduction (Fig. 3e, Supplementary Table S5). Compared to pure OPC concrete mixtures, statistical analysis using paired *t* tests (Supplementary Tables S8 and S9) demonstrates a significant reduction in damage to resources values when cement is substituted by fly ash at 10, 15, and 20 percent by weight replacements (*p* < 0.05). The figure also shows that when the concrete mix design with 20% fly ash replacement is projected at a higher compressive strength, a more significant reduction in environmental burden can be obtained. When fly ash replacement is increased from 15 to 20%, the reduction in resource degradation is substantial at the 95% confidence level *(p* < 0.05). Almost all the harm to resources in a representative 27.6 MPa concrete is caused by fossil fuel scarcity, accounting for 99% of the overall impact (Fig. 3f). At 35 MPa compressive strength, the damage to resources (USD 12.8) for a pure cement concrete shows a close result to the USD 15.8 quantified damage in the study of Chottemada et al.^60^ for concrete in the Indian context.

**Figure 4:**
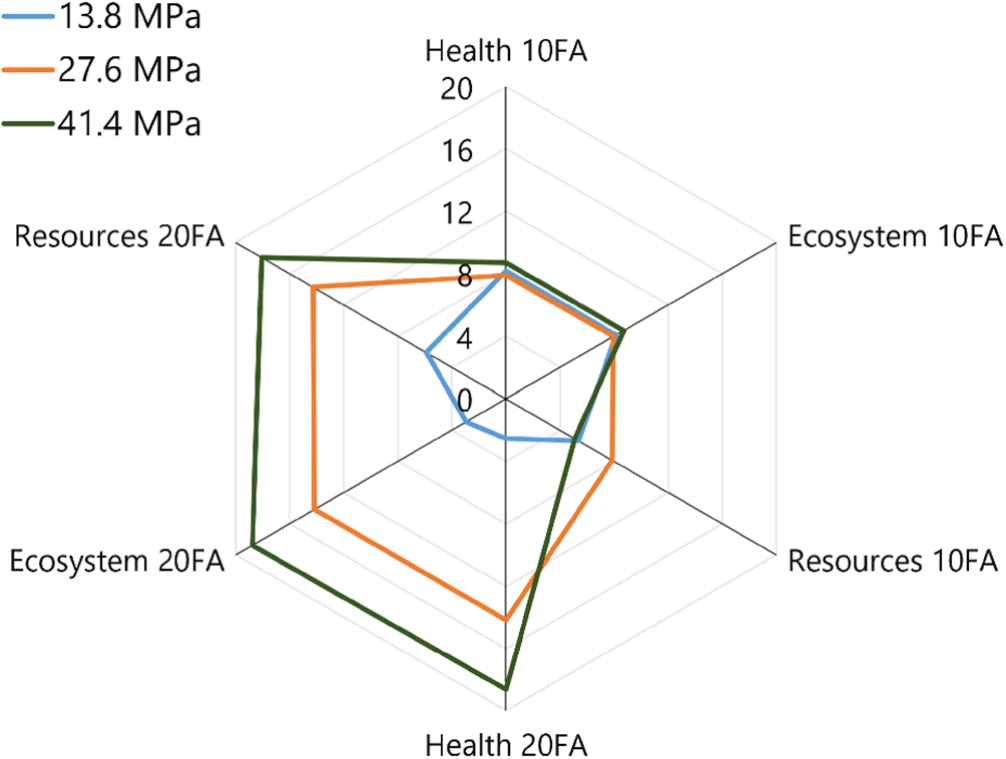
Percentage in reduction of the endpoint impacts of low (13.8 MPa), normal (27.6 MPa), and high (41.4 MPa) strength concrete with 10% and 20% fly ash replacements relative to pure cement (OPC) concrete.

The effects of employing fly ash in the three endpoint categories are shown in Fig. 4. It shows the percentage reduction in each of the three endpoint categories (damage to human health, ecosystem, and resources) for low strength (13.8 MPa), normal strength (27.6 MPa), and high strength (41.4 MPa) concrete mix designs with 10% and 20% fly ash replacements, respectively. In the figure, the greater the reduction, the better the environmental benefit of using fly ash.

For high fly ash replacement (20FA) at normal (27.6 MPa) and high-strength (41.4 MPa) concrete, there is a greater reduction in damages for all endpoint groups. However, at lower strength (13.8 MPa) concrete mix (Fig. 4), the 10% fly ash replacement mix outperforms the 20% fly ash mixture in terms of environmental performance (percentage reduction in endpoint damages). This can be explained by the fact that, in order to achieve the same 28-day compressive strength in the Philippines, concrete mixtures with higher fly ash replacements use more cement. Thus, the benefit of high fly ash replacement is not fully realized for low-strength concrete mix designs. This suggests that there is still room for improvement in the industry’s concrete mix designs by specifying long-term strength, i.e., 56 or 91 days rather than 28 days as the design strength. Furthermore, because of the slow rate of pozzolanic reaction, the beneficial impact of fly ash is most visible at later ages, i.e., longer than 28 days^61^. By comparison, for a 40 MPa concrete with 25% fly ash replacement of mass concrete mixture in Thailand, the damage to human health, ecosystem, and resources has been reduced by 29.4%, 28.2%, and 23.8%, respectively, when 56 days design strength is considered^62^.

A single score is assigned to incorporate the entire environmental burden encompassing impacts on human health, ecosystem, and resources. This score is calculated in SimaPro integrates various aspects of life cycle assessment results, including characterization, normalization, damage assessment, and weighting. The resulting single score, known as the environmental point or “Pt”, allows for the relative differences between the 31 concrete mixes to be effectively compared. The higher the environmental point, the higher the impact of the corresponding concrete mixture. By utilizing this dimensionless unit, the overall environmental impact can be readily comprehended, thus facilitating effective communication with decision-makers.

**Figure 5:**
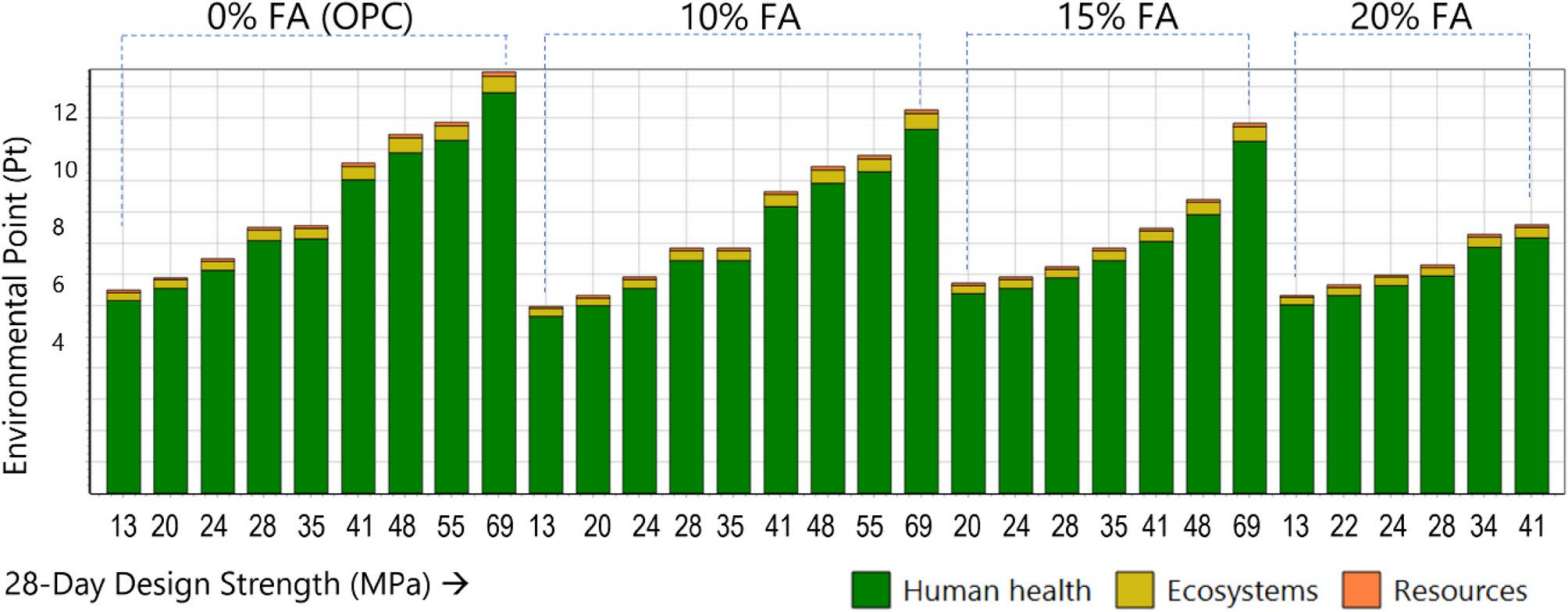
Contribution of endpoint damage indicators (human health, ecology, and resources) to the overall environmental impact of concrete with various strengths and fly ash content.

Results (Fig. 5) show that fly ash delivers more environmental benefits at higher 28-day compressive strength mix designs with higher cement replacement than at lower compressive strength mix designs. The environmental impact of concrete containing pure cement has been reduced by the following ranges when utilizing 10 to 20 percent fly ash as a replacement to cement in concrete: 2.16–18.65%, 2.55–18.65%, and 2.96–18.77% for Human Health, Ecosystem, and Resources, respectively. The greatest percentage decrease is obtained when 20% fly ash is used in high-strength concrete (41.4 MPa), whereas the smallest percentage reduction is obtained when a similar replacement is used in low-strength concrete (13.8 MPa). The more the amount of fly ash, the smaller the environmental effect, with pure cement concrete constantly producing the greatest environmental load. The results also demonstrate that health damage is the main contributing factor for all concrete mix designs. More than 90% of the environmental load is related to human health problems. This suggests that concrete production has a greater health impact than the environment and resources.

### Contribution analysis

**Figure 6:**
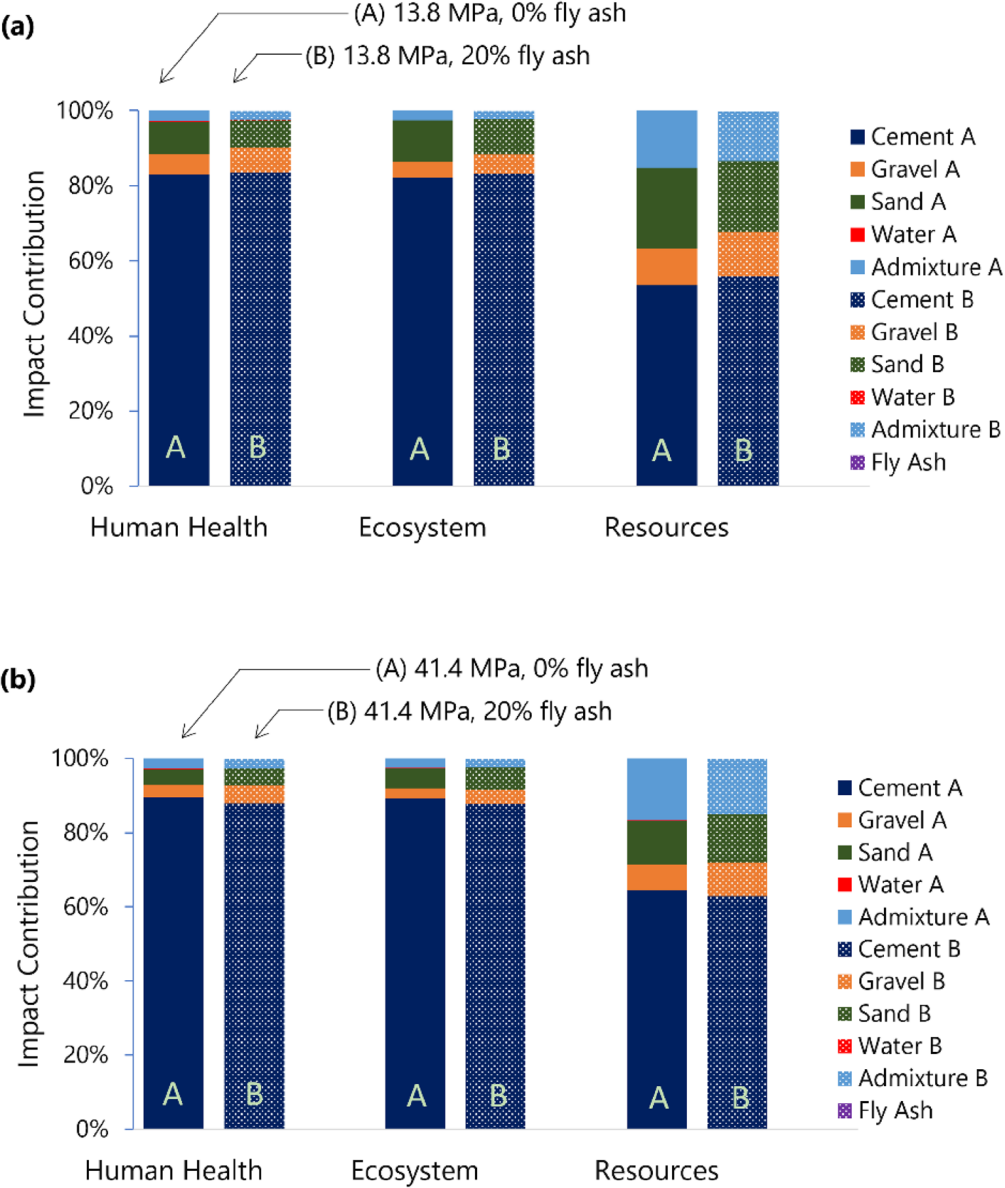
Contribution of concrete constituents to endpoint impacts of (**a**) low strength (13.8 MPa) and (**b**) high strength (41.4 MPa) concrete mixtures with 0% and 20% fly ash as cement replacements.

A contribution analysis shows that cement contributes the most to all the damage indicators. For example, for 13.8 MPa (Fig. 6a) and 41.4 MPa (Fig. 6b) concrete mixtures, this contribution is substantially more for human health (82.94%, 89.41%) and ecosystem (82.07%, 89.16%) damage than for resources (53.54%, 64.51%). A Spearman correlation analysis (Supplementary Table S10, Fig. S3) reveals a very strong relationship between cement content and ecosystem damage (*ρ* = 0.996) and human health (*ρ* = 0.996).

The admixture dosage used in the concrete mixtures reported by the ready-mix batching plants ranges from 1.00 to 1.53% by weight of the binder used. Admixture is typically used to enhance specific concrete qualities in the fresh and hardened state^63^. For all the mixtures considered, the average admixture utilized was 1.27 ± 0.17% by weight of the binder. Interestingly, even this low percentage of admixture addition provides significant damage to resources. The impact associated with resources accounts for 16.79% of 41.4 MPa concrete (Fig. 6b). A strong correlation was found using the Spearman correlation (Supplementary Table S10, Fig. S2) for all the damaged resources and the use of admixture (*ρ* = 1.00). This demonstrates that enhancing plasticizer production (or decreasing consumption) is another viable option for sustainable concrete manufacture. Few researchers have examined the environmental effects of incorporating chemicals into concrete^64^. Xing et al.^65^ stated that there is insufficient evidence to conclude the relationship between chemical admixture use and each environmental effect indicator. Conversely, Ji et al.^66^ reported a substantial impact of plasticizers. Their research quantified that the contribution to resource damage of plasticizers is 44.9% when employed in typical off-site ultra-high-performance concrete. Plasticizers are often made from petroleum feedstocks, which throughout the manufacturing process, result in the production of certain chemical components, including SO_x_ and NO_x_. These acidic gases, when emitted, will settle on land or water and modify the pH value, causing damage to the ecosystem^67^.

Another significant contributor to the damage is the aggregates (sand and gravel). The impact of aggregates is significantly higher in low-strength concrete mixtures than in high-strength concrete mixtures. For example, as shown in Fig. 6a, the contribution of aggregates for low-strength (13.8 MPa) concrete mix are 14.07%, 15.21%, and 31.18% for human health, ecosystem, and resources, respectively. This is higher than in 41.4 MPa (Fig. 6b) concrete, with contributions of aggregates 7.91% for human health, 8.39% for the ecosystem, and 22.47% for resources. The lower contribution of aggregates for higher-strength concrete is also explained by the fact that they require larger paste content (lower aggregate content) compared with lower-strength concrete mixtures. In this study, the aggregates are mining products sourced from a significant distance. One of the Philippines’ challenges is maintaining a sustainable and steady supply of aggregates. It is reported from stakeholder interviews that many batching plant operators are concerned with the future supply of aggregates in the country. This calls for sustainable practices such as the utilization of recycled aggregates. Again, the viability of recycled aggregates for applications to concrete has been reported in various studies^23,68^, but actual use in the industry has not been realized. With the country experiencing a surge in development, the industry must fully commit to its efficiency goals to ensure the optimal use of the country’s quarry resources.

### Challenges and motivations to sustainable fly ash utilization in the industry: interview results

An in-depth, semi-structured interview with the various experts provided insight into the current institutional, economic, social, and technical aspects that impede the broader adoption of waste materials in concrete construction. The major points are summarized as follows:

#### Social and institutional

The experts indicated that there is a widespread belief in the construction industry that using waste material results in a lower-quality concrete product (compared to pure cement), which has a negative impact on the performance of concrete structures. Furthermore, the current regulatory framework does not actively promote the use of these waste materials in concrete production. This is seen as a significant barrier to its widespread use. In addition, industrial by-products are commonly associated with toxicity and health risks. Sustainability is also not well-appreciated in the industry due to a lack of awareness among concrete producers, contractors, and consumers about the benefits of sustainable concrete production and the impacts of unsustainable practices.

#### Economic

One key economic impediment is an overemphasis on the initial cost of concrete construction, with the long-term benefits of waste material utilization being underappreciated. The interviewees mentioned that, to the best of their knowledge, many batching plant operators are concerned about the increased costs of integrating new technology, methods, and/or materials for large-scale concrete applications. Another major issue for certain waste by-products (i.e., slag, rice husk ash) is the country’s unstable supply. Only fly ash from coal-fired power stations is now accessible in a constant supply. The country’s high proportion of coal-fired power plants is one of the critical reasons for the widespread use of fly ash. Geographical issues are also important economically, as some waste goods must be transported by water due to the country’s archipelagic character. However, due to their durability benefits for the marine environment, the Philippines’ expansive coastlines present an outstanding potential for fly ash in marine concrete.

#### Technical

Another hindrance is a lack of technical knowledge in managing innovative ingredients for concrete mixtures. Many concrete technicians are uncertified since certification is not needed by law. More research, testing, and collaboration between industry and academia are needed. There is a need for more research to develop effective quality control methods for ensuring consistent and reliable use of fly ash in concrete production. Because codes place a high value on strength, technology is scarce for testing durability. Concrete must be exceptionally durable due to the country’s extended wet season and high humidity. Certain by-products, such as fly ash, slag, and rice husk ash, may help in this respect. Furthermore, one expert interviewee observed a lack of strong linkage between the university and the industry. While the Philippines has done multiple studies on various waste materials, most of these have been conducted in academic laboratories and have not been scaled up for industrial application.

## Opportunities for sustainable fly ash utilization in the Philippines

There is significant room for improvement in current practices in the Philippine concrete industry, with the substitution of cement by waste materials like fly ash offering enhanced sustainability. Sustainable practices should be explored not only for cement replacement but also for other concrete materials, such as aggregates and chemical admixtures. The following are identified opportunities for sustainable fly ash utilization in the Philippines based on the results of impact analysis and key stakeholder interviews:

- High fly ash replacement demonstrates greater benefits at higher concrete strength levels, providing an opportunity for improvement in the Philippine concrete industry.
- Specifying concrete strength at later ages can maximize the environmental benefits of fly ash at high replacement, particularly in the context of mass concrete.
- Consideration of hot and rainy weather conditions is crucial for handling mass concrete, emphasizing the importance of observing and controlling concrete temperatures to prevent thermal stresses and cracking.
- Further research is needed to optimize the use of fly ash in concrete production, including determining ideal mix proportions and processing techniques. Enhanced collaboration between academia and industry is essential to improve current practices.
- Strengthening laws and regulations is necessary to integrate sustainability into concrete production, with active promotion of waste materials within the industry. A supportive environment that fosters sustainability in construction, encompassing legal and economic aspects, should be established.
- Policies governing the use of cement-replacing materials should consider sustainability analysis to provide comprehensive and informed guidelines. Current regulations and standards for concrete production in the Philippines may require further development to ensure sustainability, necessitating more comprehensive and stringent policies.

## Conclusions

Results of the life cycle endpoint analysis of 31 mass concrete mixtures in the Philippines demonstrated that cement plays a significant role in causing harm to human health, the ecosystem, and resources. Global warming and fine particulate matter generation are the most significant contributors to human health damage. Terrestrial acidification, ozone formation, and land use are the other major drivers of damage related to the ecosystem. A higher fly ash replacement (20%) in normal (27.6 MPa) and high-strength (41.4 MPa) concrete leads to a greater reduction in damages across all endpoint groups, but for lower strength concrete (13.8 MPa), the 10% fly ash replacement mix performs better in terms of environmental performance. The use of admixture in mass concrete production has a significant impact on resource damage, while the incorporation of fly ash proves advantageous in reducing damage associated with human health, ecosystem, and resources.

Significant barriers to using fly ash as a cement substitute in mass concrete include institutional, economic, social, and technical dimensions. These barriers are primarily driven by the industry’s assumption that incorporating waste materials, such as fly ash, compromises concrete quality and the lack of recognition for the long-term benefits of waste material utilization in the Philippines. Furthermore, the weak connection between academia and industry hinders the implementation of state-of-the-art findings in large-scale industrial applications. It is essential to enhance laws and regulations that incentivize the use of waste materials to promote sustainable concrete production. Future research may explore the sustainability of other waste materials for mass concrete applications.

### Supplementary Information

Supplementary Information.

## Data Availability

Data will be made available on reasonable request to the corresponding author.
