# Supplementary material for: Examining the endpoint impacts, challenges, and opportunities of fly ash utilization for sustainable concrete construction
Source: Sci Rep. 2023 Oct 25;13:18254. doi: 10.1038/s41598-023-45632-z (PMC10600155; doi:10.1038/s41598-023-45632-z)
Supplement: Supplementary file 1 — Supplementary Information. [file 41598_2023_45632_MOESM1_ESM.docx]

**Supplementary Materials**

Examining the endpoint impacts, challenges, and opportunities of fly ash utilization for sustainable concrete construction

Christian Orozco^a,b^, Somnuk Tangtermsirikul^a^, Takafumi Sugiyama^c^**,** Sandhya Babel^a*^

^a^Sirindhorn International Institute of Technology, Thammasat University, P.O. Box 22, Pathum Thani 12121, Thailand

^b^Graduate School of Engineering, Hokkaido University, Japan, Sapporo 060-8628, Hokkaido, Japan

^c^Faculty of Engineering, Hokkaido University, Japan, Sapporo 060-8628, Hokkaido, Japan

**Table S1.** Interview questions to stakeholders/experts.

| 1. Could you provide insights into the current condition of concrete in the Philippines based on your expertise and experience? |
| --- |
| 2. In the concrete production industry, are waste materials currently being utilized?   - What is the rate of utilization for waste materials? - Which specific waste materials are commonly used, and is there a predominant choice? - Where are these waste materials typically sourced from? - In your opinion, what are the benefits of incorporating waste materials in concrete production? |
| 3. Are there any notable challenges associated with the utilization of waste materials in mass concrete production? |
| 4. From your perspective, what strategies or approaches can be employed to overcome the challenges related to the utilization of waste materials in concrete production? |
| 5. Are you familiar with any laws or regulations in the Philippines that promote the utilization of waste materials in concrete production? If so, could you provide further details about these regulations and their impact? |

**Table S2.** Concrete mix designs from batching plant in the Philippines

| **Mix #** | **28-day Compressive Strength, psi (MPa)** | **Cement, kg** | **Fly Ash, kg** | **Water, kg** | **Gravel, kg** | **Sand, kg** | **Admixture, kg** | **Fly Ash as Cement Replacement** | **w/b** |
| --- | --- | --- | --- | --- | --- | --- | --- | --- | --- |
| 1 | 2000 (13.8) | 260 | 0 | 130 | 950 | 995 | 3.12 | 0% | 0.50 |
| 2 | 3000 (20.7) | 280 | 0 | 150 | 1020 | 880 | 3.6 | 0% | 0.54 |
| 3 | 3500 (24.3) | 310 | 0 | 165 | 945 | 920 | 3.66 | 0% | 0.53 |
| 4 | 4000 (27.6) | 360 | 0 | 150 | 985 | 845 | 4 | 0% | 0.42 |
| 5 | 5000 (34.5) | 360 | 0 | 145 | 985 | 900 | 4.6 | 0% | 0.40 |
| 6 | 6000 (41.4) | 460 | 0 | 150 | 975 | 810 | 5.04 | 0% | 0.33 |
| 7 | 7000 (48.3) | 500 | 0 | 145 | 975 | 805 | 6 | 0% | 0.29 |
| 8 | 8000 (55.2) | 520 | 0 | 120 | 990 | 790 | 6.76 | 0% | 0.23 |
| 9 | 10000 (69.0) | 600 | 0 | 140 | 1015 | 650 | 7.8 | 0% | 0.23 |
| 10 | 2000 (13.8) | 234 | 26 | 130 | 950 | 995 | 3.12 | 10% | 0.50 |
| 11 | 3000 (20.7) | 252 | 28 | 150 | 1020 | 880 | 3.6 | 10% | 0.54 |
| 12 | 3500 (24.3) | 279 | 31 | 155 | 990 | 950 | 3.72 | 10% | 0.50 |
| 13 | 4000 (27.6) | 324 | 36 | 145 | 965 | 895 | 4.68 | 10% | 0.40 |
| 14 | 5000 (34.5) | 324 | 36 | 145 | 985 | 900 | 4.6 | 10% | 0.40 |
| 15 | 6000 (41.4) | 414 | 46 | 155 | 970 | 800 | 5.52 | 10% | 0.34 |
| 16 | 7000 (48.3) | 450 | 50 | 130 | 855 | 895 | 6.5 | 10% | 0.26 |
| 17 | 8000 (55.2) | 468 | 52 | 120 | 990 | 790 | 6.76 | 10% | 0.23 |
| 18 | 10000 (69.0) | 540 | 60 | 140 | 1015 | 650 | 7.8 | 10% | 0.23 |
| 19 | 3000 (20.7) | 272 | 48 | 165 | 945 | 925 | 3.52 | 15% | 0.52 |
| 20 | 3500 (24.3) | 281 | 49 | 165 | 945 | 920 | 3.66 | 15% | 0.50 |
| 21 | 4000 (27.6) | 298 | 53 | 165 | 975 | 875 | 3.9 | 15% | 0.47 |
| 22 | 5000 (34.5) | 323 | 57 | 140 | 985 | 885 | 4.9 | 15% | 0.37 |
| 23 | 6000 (41.4) | 357 | 63 | 150 | 975 | 810 | 5.04 | 15% | 0.36 |
| 24 | 7000 (48.3) | 400 | 70 | 145 | 975 | 805 | 6 | 15% | 0.31 |
| 25 | 10000 (69.0) | 519 | 91 | 145 | 1050 | 625 | 7.93 | 15% | 0.24 |
| 26 | 2000 (13.8) | 256 | 64 | 165 | 1078 | 830 | 2.56 | 20% | 0.52 |
| 27 | 3000 (20.7) | 272 | 68 | 165 | 1078 | 814 | 2.72 | 20% | 0.49 |
| 28 | 3500 (24.3) | 288 | 72 | 165 | 1078 | 798 | 2.88 | 20% | 0.46 |
| 29 | 4000 (27.6) | 304 | 76 | 165 | 1078 | 782 | 3.04 | 20% | 0.43 |
| 30 | 5000 (34.5) | 352 | 88 | 165 | 1078 | 735 | 3.52 | 20% | 0.38 |
| 31 | 6000 (41.4) | 368 | 92 | 165 | 1078 | 719 | 3.68 | 20% | 0.36 |


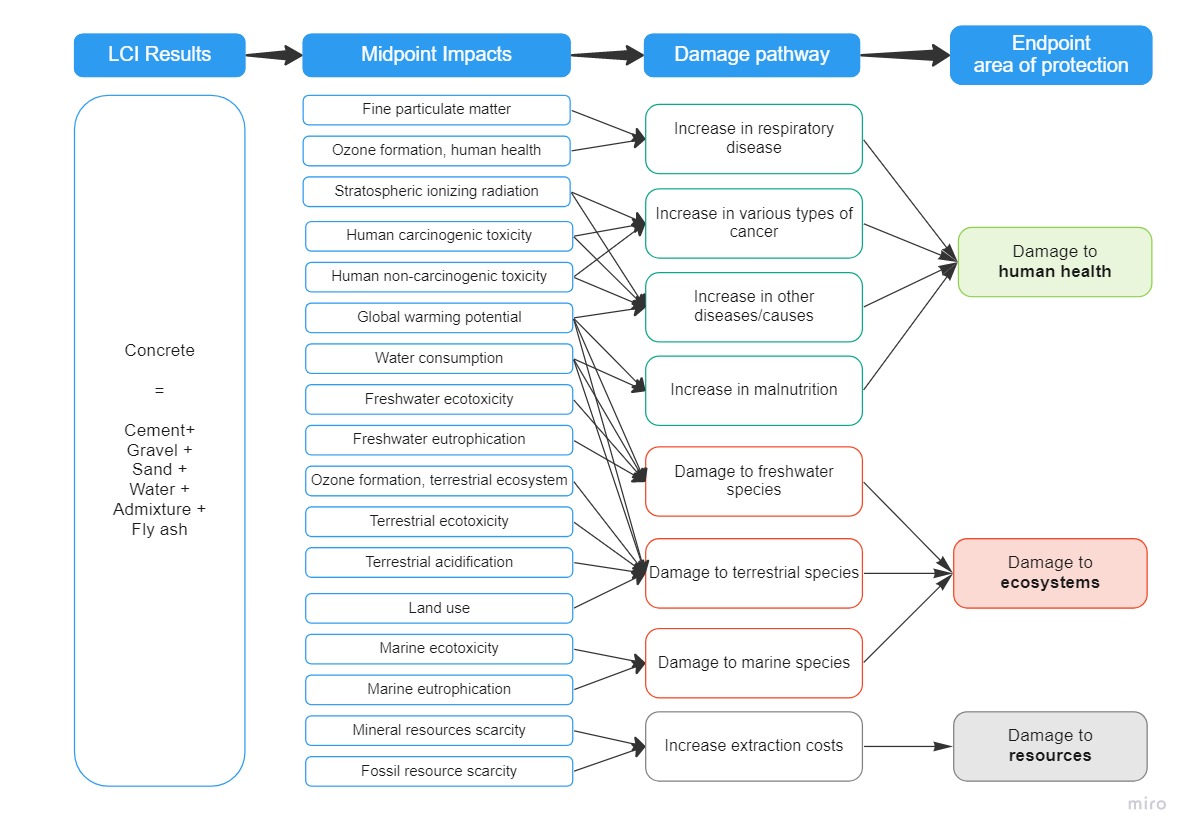


**Fig. S1.**  Overview of the impact categories that are covered in the ReCiPe 2016 method and their relation to the areas of protection (Huijbregts et al, 2017).

**Table S3.** Human health (H) impact of various concrete mix and corresponding reduction percentage

|  | Human health (Disability Adjusted Life Years) | | | |  | Reduction, % | | |
| --- | --- | --- | --- | --- | --- | --- | --- | --- |
| Strength (MPa) | H OPC | H 10FA | H 15FA | H 20FA |  | OPC vs 10FA | OPC vs 15FA | OPC vs 20FA |
| 13.79 | 3.70E-04 | 3.39E-04 | - | 3.60E-04 |  | 8.18% | - | 2.56% |
| 20.68 | 3.92E-04 | 3.60E-04 | 3.82E-04 | 3.79E-04 |  | 8.30% | 2.55% | 3.46% |
| 24.1 | 4.27E-04 | 3.93E-04 | 3.93E-04 | 3.97E-04 |  | 7.97% | 7.94% | 6.95% |
| 27.58 | 4.84E-04 | 4.46E-04 | 4.13E-04 | 4.16E-04 |  | 7.94% | 14.79% | 14.15% |
| 34.47 | 4.88E-04 | 4.46E-04 | 4.45E-04 | 4.71E-04 |  | 8.58% | 8.74% | 3.44% |
| 41.37 | 6.02E-04 | 5.50E-04 | 4.83E-04 | 4.90E-04 |  | 8.69% | 19.85% | 18.65% |
| 48.26 | 6.54E-04 | 5.95E-04 | 5.35E-04 | - |  | 8.98% | 18.14% | - |
| 55.16 | 6.77E-04 | 6.16E-04 | - | - |  | 8.93% | - | - |
| 68.95 | 7.69E-04 | 6.99E-04 | 6.75E-04 | - |  | 9.08% | 12.22% | - |

**Table S4.** Ecosystem (E) impact of various concrete mix and corresponding reduction percentage

|  | Ecosystem (species-yr) | | | |  | Reduction, % | | |
| --- | --- | --- | --- | --- | --- | --- | --- | --- |
| Strength (MPa) | E OPC | E 10FA | E 15FA | E 20FA |  | OPC vs 10FA | OPC vs 15FA | OPC vs 20FA |
| 13.79 | 9.43E-07 | 8.66E-07 | - | 9.15E-07 |  | 8.14% | - | 2.97% |
| 20.68 | 9.97E-07 | 9.14E-07 | 9.74E-07 | 9.62E-07 |  | 8.29% | 2.33% | 3.55% |
| 24.1 | 1.09E-06 | 1.00E-06 | 1.00E-06 | 1.01E-06 |  | 7.92% | 7.90% | 7.21% |
| 27.58 | 1.23E-06 | 1.13E-06 | 1.05E-06 | 1.05E-06 |  | 7.86% | 14.71% | 14.26% |
| 34.47 | 1.24E-06 | 1.13E-06 | 1.13E-06 | 1.19E-06 |  | 8.56% | 8.75% | 3.68% |
| 41.37 | 1.53E-06 | 1.40E-06 | 1.22E-06 | 1.24E-06 |  | 8.71% | 19.86% | 18.77% |
| 48.26 | 1.66E-06 | 1.51E-06 | 1.36E-06 | - |  | 8.78% | 18.12% | - |
| 55.16 | 1.72E-06 | 1.56E-06 | - | - |  | 8.93% | - | - |
| 68.95 | 1.95E-06 | 1.77E-06 | 1.71E-06 | - |  | 9.09% | 12.28% | - |

**Table S5.** Resources (R) impact of various concrete mix and corresponding reduction percentage

|  | Resources (USD 2013) | | | |  | Reduction, % | | |
| --- | --- | --- | --- | --- | --- | --- | --- | --- |
| Strength (MPa) | R OPC | R 10FA | R 15FA | R 20FA |  | OPC vs 10FA | OPC vs 15FA | OPC vs 20FA |
| 13.79 | 10.18104 | 9.635488 | - | 9.581625 |  | 5.36% | - | 5.89% |
| 20.68 | 10.65452 | 10.06701 | 10.4528 | 9.954085 |  | 5.51% | 1.89% | 6.57% |
| 24.1 | 11.31139 | 10.80102 | 10.69579 | 10.32655 |  | 4.51% | 5.44% | 8.71% |
| 27.58 | 12.38876 | 12.0523 | 11.0968 | 10.69901 |  | 2.72% | 10.43% | 13.64% |
| 34.47 | 12.80006 | 12.04468 | 12.13084 | 11.81854 |  | 5.90% | 5.23% | 7.67% |
| 41.37 | 14.87436 | 14.11732 | 12.72735 | 12.191 |  | 5.09% | 14.43% | 18.04% |
| 48.26 | 16.52686 | 15.42145 | 14.07022 | - |  | 6.69% | 14.86% | - |
| 55.16 | 16.92179 | 15.8332 | - | - |  | 6.43% | - | - |
| 68.95 | 18.8088 | 17.54985 | 17.15199 | - |  | 6.69% | 8.81% | - |

**Table S6**. Results of paired t-test for comparison (p-values) for human health impacts

|  | OPC | 10FA | 15FA | 20FA |
| --- | --- | --- | --- | --- |
| OPC | - |  |  |  |
| 10FA | 0.000 | - |  |  |
| 15FA | 0.005 | 0.112 | - |  |
| 20FA | 0.054 | 0.814 | 0.211 | - |

**Table S7**. Results of paired t-test for comparison (p-values) for ecosystem impacts

|  | OPC | 10FA | 15FA | 20FA |
| --- | --- | --- | --- | --- |
| OPC | - |  |  |  |
| 10FA | 0.000 | - |  |  |
| 15FA | 0.005 | 0.114 | - |  |
| 20FA | 0.050 | 0.761 | 0.271 | - |

**Table S8**. Results of paired t-test for comparison (p-values) for resources impacts

|  | OPC | 10FA | 15FA | 20FA |
| --- | --- | --- | --- | --- |
| OPC | - |  |  |  |
| 10FA | 0.000 | - |  |  |
| 15FA | 0.007 | 0.094 | - |  |
| 20FA | 0.011 | 0.079 | 0.001 | - |

**Table S9**. Paired-t test calculations for comparison of human health (H), ecosystem (E), and resources (R) impacts for various concrete mix designs with varying fly ash content.

**Table S9a.** Paired T-Test and CI: H OPC, H 10FA

Descriptive Statistics

| Sample | N | Mean | StDev | SE Mean |
| --- | --- | --- | --- | --- |
| H OPC | 9 | 0.000540 | 0.000140 | 0.000047 |
| H 10FA | 9 | 0.000494 | 0.000126 | 0.000042 |

Estimation for Paired Difference

| Mean | StDev | SE Mean | 95% CI for μ_difference |
| --- | --- | --- | --- |
| 0.000046 | 0.000014 | 0.000005 | (0.000036, 0.000057) |

*µ_difference: population mean of (H OPC - H 10FA)*

Test

| Null hypothesis | | H₀: μ_difference = 0 |
| --- | --- | --- |
| Alternative hypothesis | | H₁: μ_difference ≠ 0 |
| T-Value | P-Value |  |
| 9.80 | 0.000 |  |

**Table S9b.** Paired T-Test and CI: H OPC, H 15FA

Descriptive Statistics

| Sample | N | Mean | StDev | SE Mean |
| --- | --- | --- | --- | --- |
| H OPC | 7 | 0.000545 | 0.000135 | 0.000051 |
| H 15FA | 7 | 0.000475 | 0.000103 | 0.000039 |

Estimation for Paired Difference

| Mean | StDev | SE Mean | 95% CI for μ_difference |
| --- | --- | --- | --- |
| 0.000070 | 0.000043 | 0.000016 | (0.000030, 0.000110) |

*µ_difference: population mean of (H OPC - H 15FA)*

Test

| Null hypothesis | | H₀: μ_difference = 0 |
| --- | --- | --- |
| Alternative hypothesis | | H₁: μ_difference ≠ 0 |
| T-Value | P-Value |  |
| 4.32 | 0.005 |  |

**Table S9c.** Paired T-Test and CI: H OPC, H 20FA

Descriptive Statistics

| Sample | N | Mean | StDev | SE Mean |
| --- | --- | --- | --- | --- |
| H OPC | 6 | 0.000461 | 0.000084 | 0.000034 |
| H 20FA | 6 | 0.000419 | 0.000052 | 0.000021 |

Estimation for Paired Difference

| Mean | StDev | SE Mean | 95% CI for μ_difference |
| --- | --- | --- | --- |
| 0.000042 | 0.000041 | 0.000017 | (-0.000001, 0.000084) |

*µ_difference: population mean of (H OPC - H 20FA)*

Test

| Null hypothesis | | H₀: μ_difference = 0 |
| --- | --- | --- |
| Alternative hypothesis | | H₁: μ_difference ≠ 0 |
| T-Value | P-Value |  |
| 2.51 | 0.054 |  |

**Table S9d.** Paired T-Test and CI: H 10FA, H 15FA

Descriptive Statistics

| Sample | N | Mean | StDev | SE Mean |
| --- | --- | --- | --- | --- |
| H 10FA | 7 | 0.000498 | 0.000121 | 0.000046 |
| H 15FA | 7 | 0.000475 | 0.000103 | 0.000039 |

Estimation for Paired Difference

| Mean | StDev | SE Mean | 95% CI for μ_difference |
| --- | --- | --- | --- |
| 0.000023 | 0.000033 | 0.000012 | (-0.000007, 0.000054) |

*µ_difference: population mean of (H 10FA - H 15FA)*

Test

| Null hypothesis | | H₀: μ_difference = 0 |
| --- | --- | --- |
| Alternative hypothesis | | H₁: μ_difference ≠ 0 |
| T-Value | P-Value |  |
| 1.86 | 0.112 |  |

**Table S9e.** Paired T-Test and CI: H 10FA, H 20FA

Descriptive Statistics

| Sample | N | Mean | StDev | SE Mean |
| --- | --- | --- | --- | --- |
| H 10FA | 6 | 0.000422 | 0.000076 | 0.000031 |
| H 20FA | 6 | 0.000419 | 0.000052 | 0.000021 |

Estimation for Paired Difference

| Mean | StDev | SE Mean | 95% CI for μ_difference |
| --- | --- | --- | --- |
| 0.000003 | 0.000034 | 0.000014 | (-0.000033, 0.000039) |

*µ_difference: population mean of (H 10FA - H 20FA)*

Test

| Null hypothesis | | H₀: μ_difference = 0 |
| --- | --- | --- |
| Alternative hypothesis | | H₁: μ_difference ≠ 0 |
| T-Value | P-Value |  |
| 0.25 | 0.814 |  |

**Table S9f.** Paired T-Test and CI: H 15FA, H 20FA

Descriptive Statistics

| Sample | N | Mean | StDev | SE Mean |
| --- | --- | --- | --- | --- |
| H 15FA | 5 | 0.000423 | 0.000041 | 0.000018 |
| H 20FA | 5 | 0.000431 | 0.000048 | 0.000021 |

Estimation for Paired Difference

| Mean | StDev | SE Mean | 95% CI for μ_difference |
| --- | --- | --- | --- |
| -0.000007 | 0.000011 | 0.000005 | (-0.000021, 0.000006) |

*µ_difference: population mean of (H 15FA - H 20FA)*

Test

| Null hypothesis | | H₀: μ_difference = 0 |
| --- | --- | --- |
| Alternative hypothesis | | H₁: μ_difference ≠ 0 |
| T-Value | P-Value |  |
| -1.49 | 0.211 |  |

**Table S9g.** Paired T-Test and CI: E OPC, E 10FA

Descriptive Statistics

| Sample | N | Mean | StDev | SE Mean |
| --- | --- | --- | --- | --- |
| E OPC | 9 | 0.000001 | 0.000000 | 0.000000 |
| E 10FA | 9 | 0.000001 | 0.000000 | 0.000000 |

Estimation for Paired Difference

| Mean | StDev | SE Mean | 95% CI for μ_difference |
| --- | --- | --- | --- |
| 0.000000 | 0.000000 | 0.000000 | (0.000000, 0.000000) |

*µ_difference: population mean of (E OPC - E 10FA)*

Test

| Null hypothesis | | H₀: μ_difference = 0 |
| --- | --- | --- |
| Alternative hypothesis | | H₁: μ_difference ≠ 0 |
| T-Value | P-Value |  |
| 9.83 | 0.000 |  |

**Table S9h.** Paired T-Test and CI: E OPC, E 15FA

Descriptive Statistics

| Sample | N | Mean | StDev | SE Mean |
| --- | --- | --- | --- | --- |
| E OPC | 7 | 0.000001 | 0.000000 | 0.000000 |
| E 15FA | 7 | 0.000001 | 0.000000 | 0.000000 |

Estimation for Paired Difference

| Mean | StDev | SE Mean | 95% CI for μ_difference |
| --- | --- | --- | --- |
| 0.000000 | 0.000000 | 0.000000 | (0.000000, 0.000000) |

*µ_difference: population mean of (E OPC - E 15FA)*

Test

| Null hypothesis | | H₀: μ_difference = 0 |
| --- | --- | --- |
| Alternative hypothesis | | H₁: μ_difference ≠ 0 |
| T-Value | P-Value |  |
| 4.29 | 0.005 |  |

**Table S9i.** Paired T-Test and CI: E OPC, E 20FA

Descriptive Statistics

| Sample | N | Mean | StDev | SE Mean |
| --- | --- | --- | --- | --- |
| E OPC | 6 | 0.000001 | 0.000000 | 0.000000 |
| E 20FA | 6 | 0.000001 | 0.000000 | 0.000000 |

Estimation for Paired Difference

| Mean | StDev | SE Mean | 95% CI for μ_difference |
| --- | --- | --- | --- |
| 0.000000 | 0.000000 | 0.000000 | (0.000000, 0.000000) |

*µ_difference: population mean of (E OPC - E 20FA)*

Test

| Null hypothesis | | H₀: μ_difference = 0 |
| --- | --- | --- |
| Alternative hypothesis | | H₁: μ_difference ≠ 0 |
| T-Value | P-Value |  |
| 2.58 | 0.050 |  |

**Table S9j.** Paired T-Test and CI: E 10FA, E 15FA

Descriptive Statistics

| Sample | N | Mean | StDev | SE Mean |
| --- | --- | --- | --- | --- |
| E 10FA | 7 | 0.000001 | 0.000000 | 0.000000 |
| E 15FA | 7 | 0.000001 | 0.000000 | 0.000000 |

Estimation for Paired Difference

| Mean | StDev | SE Mean | 95% CI for μ_difference |
| --- | --- | --- | --- |
| 0.000000 | 0.000000 | 0.000000 | (-0.000000, 0.000000) |

*µ_difference: population mean of (E 10FA - E 15FA)*

Test

| Null hypothesis | | H₀: μ_difference = 0 |
| --- | --- | --- |
| Alternative hypothesis | | H₁: μ_difference ≠ 0 |
| T-Value | P-Value |  |
| 1.85 | 0.114 |  |

**Table S9k.** Paired T-Test and CI: E 10FA, E 20FA

Descriptive Statistics

| Sample | N | Mean | StDev | SE Mean |
| --- | --- | --- | --- | --- |
| E 10FA | 6 | 0.000001 | 0.000000 | 0.000000 |
| E 20FA | 6 | 0.000001 | 0.000000 | 0.000000 |

Estimation for Paired Difference

| Mean | StDev | SE Mean | 95% CI for μ_difference |
| --- | --- | --- | --- |
| 0.000000 | 0.000000 | 0.000000 | (-0.000000, 0.000000) |

*µ_difference: population mean of (E 10FA - E 20FA)*

Test

| Null hypothesis | | H₀: μ_difference = 0 |
| --- | --- | --- |
| Alternative hypothesis | | H₁: μ_difference ≠ 0 |
| T-Value | P-Value |  |
| 0.32 | 0.761 |  |

**Table S9l.** Paired T-Test and CI: E 15FA, E 20FA

Descriptive Statistics

| Sample | N | Mean | StDev | SE Mean |
| --- | --- | --- | --- | --- |
| E 15FA | 5 | 0.000001 | 0.000000 | 0.000000 |
| E 20FA | 5 | 0.000001 | 0.000000 | 0.000000 |

Estimation for Paired Difference

| Mean | StDev | SE Mean | 95% CI for μ_difference |
| --- | --- | --- | --- |
| -0.000000 | 0.000000 | 0.000000 | (-0.000000, 0.000000) |

*µ_difference: population mean of (E 15FA - E 20FA)*

Test

| Null hypothesis | | H₀: μ_difference = 0 |
| --- | --- | --- |
| Alternative hypothesis | | H₁: μ_difference ≠ 0 |
| T-Value | P-Value |  |
| -1.28 | 0.271 |  |

**Table S9m.** Paired T-Test and CI: R OPC, R 10FA

Descriptive Statistics

| Sample | N | Mean | StDev | SE Mean |
| --- | --- | --- | --- | --- |
| R OPC | 9 | 13.83 | 3.07 | 1.02 |
| R 10FA | 9 | 13.06 | 2.79 | 0.93 |

Estimation for Paired Difference

| Mean | StDev | SE Mean | 95% CI for μ_difference |
| --- | --- | --- | --- |
| 0.772 | 0.315 | 0.105 | (0.530, 1.014) |

*µ_difference: population mean of (R OPC - R 10FA)*

Test

| Null hypothesis | | H₀: μ_difference = 0 |
| --- | --- | --- |
| Alternative hypothesis | | H₁: μ_difference ≠ 0 |
| T-Value | P-Value |  |
| 7.36 | 0.000 |  |

**Table S9n.** Paired T-Test and CI: R OPC, R 15FA

Descriptive Statistics

| Sample | N | Mean | StDev | SE Mean |
| --- | --- | --- | --- | --- |
| R OPC | 7 | 13.91 | 2.96 | 1.12 |
| R 15FA | 7 | 12.62 | 2.37 | 0.89 |

Estimation for Paired Difference

| Mean | StDev | SE Mean | 95% CI for μ_difference |
| --- | --- | --- | --- |
| 1.291 | 0.842 | 0.318 | (0.512, 2.070) |

*µ_difference: population mean of (R OPC - R 15FA)*

Test

| Null hypothesis | | H₀: μ_difference = 0 |
| --- | --- | --- |
| Alternative hypothesis | | H₁: μ_difference ≠ 0 |
| T-Value | P-Value |  |
| 4.06 | 0.007 |  |

**Table S9o.** Paired T-Test and CI: R OPC, R 20FA

Descriptive Statistics

| Sample | N | Mean | StDev | SE Mean |
| --- | --- | --- | --- | --- |
| R OPC | 6 | 12.035 | 1.711 | 0.699 |
| R 20FA | 6 | 10.762 | 1.039 | 0.424 |

Estimation for Paired Difference

| Mean | StDev | SE Mean | 95% CI for μ_difference |
| --- | --- | --- | --- |
| 1.273 | 0.789 | 0.322 | (0.445, 2.101) |

*µ_difference: population mean of (R OPC - R 20FA)*

Test

| Null hypothesis | | H₀: μ_difference = 0 |
| --- | --- | --- |
| Alternative hypothesis | | H₁: μ_difference ≠ 0 |
| T-Value | P-Value |  |
| 3.95 | 0.011 |  |

**Table S9p.** Paired T-Test and CI: R 10FA, R 15FA

Descriptive Statistics

| Sample | N | Mean | StDev | SE Mean |
| --- | --- | --- | --- | --- |
| R 10FA | 7 | 13.15 | 2.67 | 1.01 |
| R 15FA | 7 | 12.62 | 2.37 | 0.89 |

Estimation for Paired Difference

| Mean | StDev | SE Mean | 95% CI for μ_difference |
| --- | --- | --- | --- |
| 0.533 | 0.708 | 0.268 | (-0.123, 1.188) |

*µ_difference: population mean of (R 10FA - R 15FA)*

Test

| Null hypothesis | | H₀: μ_difference = 0 |
| --- | --- | --- |
| Alternative hypothesis | | H₁: μ_difference ≠ 0 |
| T-Value | P-Value |  |
| 1.99 | 0.094 |  |

**Table S9q.** Paired T-Test and CI: R 10FA, R 20FA

Descriptive Statistics

| Sample | N | Mean | StDev | SE Mean |
| --- | --- | --- | --- | --- |
| R 10FA | 6 | 11.453 | 1.641 | 0.670 |
| R 20FA | 6 | 10.762 | 1.039 | 0.424 |

Estimation for Paired Difference

| Mean | StDev | SE Mean | 95% CI for μ_difference |
| --- | --- | --- | --- |
| 0.691 | 0.770 | 0.315 | (-0.117, 1.500) |

*µ_difference: population mean of (R 10FA - R 20FA)*

Test

| Null hypothesis | | H₀: μ_difference = 0 |
| --- | --- | --- |
| Alternative hypothesis | | H₁: μ_difference ≠ 0 |
| T-Value | P-Value |  |
| 2.20 | 0.079 |  |

**Table S9r.** Paired T-Test and CI: R 15FA, R 20FA

Descriptive Statistics

| Sample | N | Mean | StDev | SE Mean |
| --- | --- | --- | --- | --- |
| R 15FA | 5 | 11.421 | 0.972 | 0.435 |
| R 20FA | 5 | 10.998 | 0.965 | 0.432 |

Estimation for Paired Difference

| Mean | StDev | SE Mean | 95% CI for μ_difference |
| --- | --- | --- | --- |
| 0.4229 | 0.0927 | 0.0414 | (0.3078, 0.5380) |

*µ_difference: population mean of (R 15FA - R 20FA)*

Test

| Null hypothesis | | H₀: μ_difference = 0 |
| --- | --- | --- |
| Alternative hypothesis | | H₁: μ_difference ≠ 0 |
| T-Value | P-Value |  |
| 10.20 | 0.001 |  |

**Table S10.** Spearman correlation of human health (H), ecosystem (E), and resources (R) impacts to various concrete ingredients for cement-only mixtures (OPC).

|  | **H OPC** | **E OPC** | **R OPC** | **Cement** | **Water** | **Gravel** | **Sand** |
| --- | --- | --- | --- | --- | --- | --- | --- |
| Cement | 0.996 | 0.996 | 0.996 |  |  |  |  |
| Water | -0.392 | -0.392 | -0.392 | -0.372 |  |  |  |
| Gravel | 0.353 | 0.353 | 0.353 | 0.354 | -0.249 |  |  |
| Sand | -0.917 | -0.917 | -0.917 | -0.937 | 0.341 | -0.546 |  |
| Admixture | 1.000 | 1.000 | 1.000 | 0.996 | -0.392 | 0.353 | -0.917 |


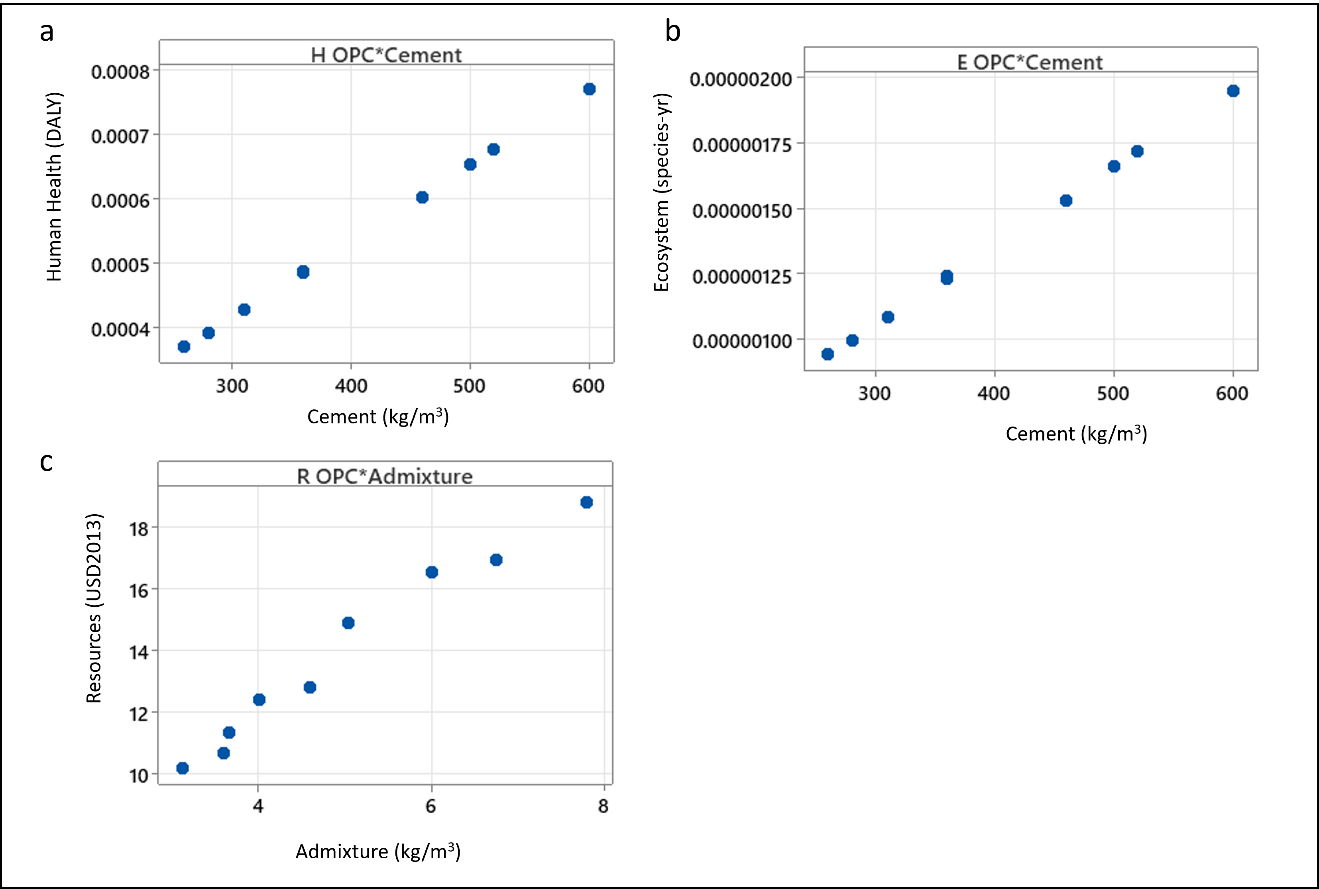


**Fig. S2.**  Scatterplot of (a) human health vs cement content, (b) ecosystem vs cement content, and (c) resources vs admixture
